# Do African-American men need separate prostate cancer screening guidelines?

**DOI:** 10.1186/s12894-016-0137-7

**Published:** 2016-05-10

**Authors:** Divya Shenoy, Satyaseelan Packianathan, Allen M. Chen, Srinivasan Vijayakumar

**Affiliations:** University of Mississippi Medical School, Jackson, MS USA; University of Mississippi Medical Center, 2500 North State Street, 39216-4505 Jackson, MS USA; University of California, Los Angeles-David Geffen School of Medicine, Los Angeles, California USA; UMMC Cancer Institute, Department of Radiation Oncology, 2500 North State Street, 39216-4505 Jackson, MS USA

**Keywords:** Prostate cancer, African Americans, Screening guidelines

## Abstract

**Background:**

In 2012, the United States Preventative Services Task Force issued new guidelines recommending that male U.S. residents, irrespective of race, no longer be screened for prostate cancer. In African American men, the incidence of prostate cancer is almost 60 % higher and the mortality rate is two to three times greater than in Caucasians. The purpose of this study is to reduce African American men's prostate cancer burden by demonstrating they need separate screening guidelines.

**Methods:**

We performed a PubMed search using the keywords: African American, Prostate cancer, Outcomes, Molecular markers, Prostate-specific Antigen velocity, PSA density, and to derive data relevant to our hypothesis.

**Results:**

In our literature review, we identified several aspects of prostate cancer that are different in Caucasian and African American men. These included prostate cancer incidence and outcome, the clinical course of the disease, serum PSA levels, genetic differences, and social barriers. It's also important to note that the USPSTF guidelines were based on two studies, one of which reported that only 4 % of its participants were African American. The other did not report demographic information, but used participants from seven European countries with small African American populations.

**Conclusion:**

Given the above, we conclude that separate prostate cancer screening guidelines are greatly necessary to help save the lives of African Americans.

## Background

Prostate cancer represents the most common male visceral cancer in the United States. The estimated number of annual new diagnoses and mortality in 2015 is 220,800 and 27,540, respectively [1]. The life time risk of a diagnosis of prostate cancer is 15.9 % while the life time risk of death is 2.8 % [2]. In African-American men, however, the incidence of prostate cancer is almost 60 % higher and the mortality rate is two- to three-times greater than that of Caucasian men. These numbers have remained remarkably constant for more than 20 years [3].

A commonly held perception about prostate cancer in the lay public and even within the scientific community is that prostate cancer is generally an indolent disease and therefore not as deadly because individuals who are diagnosed with prostate cancer often die of other causes unrelated to the cancer. Although this view may be true to a certain extent when the at-risk U.S. population is taken as a whole, it is our contention that it should not apply to all prostate cancer patients, especially to those of African-American descent. Because of differences in the natural course of the disease and because of social issues that differentially impact African Americans, many of these patients may face a greater health burden than their Caucasian counterparts once they have been diagnosed [4–6]. Hence, a diagnosis that is made as early as possible in this population may improve overall prostate cancer outcomes. In this regard, we believe that the current prostate cancer screening guidelines promulgated by the U.S. Preventative Services Task Force (USPSTF) do not adequately take into account ethnic/racial differences in the impact of prostate cancer on the population. Separate screening guidelines for African American men would facilitate earlier diagnosis and thereby reduce their mortality rate from the disease.

### Hypothesis

African American men need separate screening guidelines to help decrease their prostate cancer burden.

## Methods

We performed a “Pubmed search” using the following keywords:

- African American
- Prostate cancer
- Outcomes
- Molecular markers
- Prostate-specific antigen (PSA) velocity
- PSA density

We did not place any time restrictions. Specifically, we used the word “African American” and then one of the other five words listed above to create a total of four combinations total. The articles derived from the initial search were culled to identify peer-reviewed publications that were relevant to the clinical, social, and health policy aspects of the diagnosis and treatment of prostate cancer in African American men. These manuscripts were then analyzed closely to derive other sources of information and data that were relevant to our hypothesis.

In our review of the literature, we identified several aspects of prostate cancer that are different in Caucasian and African-American males. These included prostate cancer incidence and outcomes, the clinical course of the disease itself, serum PSA levels, and social barriers/behaviors. These are discussed in more detail in subsequent sections below.

## Results/Discussion

In 2012, the USPSTF issued new guidelines recommending that U.S. male residents, irrespective of their race, no longer be screened for prostate cancer using PSA. In early 2015, the American Cancer Society recommended men who are considering being screened for prostate cancer should make an informed decision only after discussing their prostate cancer specific risk factors and general clinical situation, considering variables such as overall health, performance status, and the presence or absence of medical comorbidities, with their physician. There are several differences between African American and Caucasian men that make such guidelines ineffective for African Americans.

### Incidence of prostate cancer among African Americans

The incidence of prostate cancer among African American males is higher than in any ethnic/racial group in the United States. In fact, worldwide, those men with West African ancestry have a disproportionately larger burden of prostate cancer [3]. In the United States, the incidence of prostate cancer in African American males has been 60 % higher than the incidence in Caucasians [3]. Furthermore, there are sociological aspects that adversely affect the incidence of prostate cancer in African American males. These will be discussed in sections below.

### Clinical course of prostate cancer in African Americans

The course of prostate cancer has been shown to be different in African American men; for instance, prostate cancer volume is greater in African American men and advanced metastatic prostate cancer occurs at a 4:1 ratio in black and white men, respectively [7]. While clinical characteristics do not differ by race at an early age, prostate cancer transforms earlier in black men (from indolent to aggressive). Therefore, one can surmise that the growth or transformation rate of prostate cancer is greater in black men. Because of this augmented growth rate, African American men likely present at a later stage of disease than Caucasians at a similar age. Additionally, among African American patients who had prostate biopsies without histological evidence of prostate cancer, their PSA values were about 1.8-fold higher than those in whites, even when controlling for prostate volume [8]. Genetically, there seem to be differences between African American and white males as well. For example, it has been suggested that African American men may be more susceptible to prostate cancer because of differences in the expression of the androgen receptor genes [9]. Additionally, several risk-associated single nucleotide polymorphisms (SNPs), appear to be differentially overexpressed in African American men compared to control subjects [10].

### Social Differences in the African American Population

African American men face several social barriers that Caucasian men may not, contributing further to their risk of poorer prognoses and outcomes. For example, when comparing men over the age of 50, only 81 % of African Americans are likely to have health insurance compared to nearly 90 % of Caucasians. This difference is statistically significant [4]. African American men have been noted to be less likely to be treated for prostate cancer when compared to white patients with a similar stage of disease [6]. Financial barriers associated with a lower socioeconomic status (SES) are also a major contributor to racial disparities in prostate cancer outcomes [10, 11]. Nonfinancial related barriers such as poor health seeking behaviors often can delay a prostate cancer diagnosis in African American men [5]. Thus, an inherent fear of a cancer diagnosis coupled with a mistrust of the health care system at large, can significantly affect the outcome of African-American men with prostate cancer [5].

Diets high in fat content have been associated with an increased risk of prostate cancer. Specifically, a diet higher in saturated fats is positively correlated to an increased risk of prostate cancer. When considering Caucasians, Asians, and African Americans, African Americans generally tend to consume more saturated fats and a greater number of calories in their daily diet [9]. This dietary discrepancy alone may account for almost 10 % of the difference in prostate cancer incidence between Caucasian and African American men [12].

### PSA levels are higher among African Americans

In general, African American men present with higher PSA values when compared to white men [13]. Several studies have shown that African American men with nonmetastatic prostate cancer have higher serum PSA levels at diagnosis than Caucasian men with nonmetastatic prostate cancer, suggesting that perhaps African Americans have a higher tumor cell burden [13, 14]. Furthermore, African American men with or without evidence for prostate cancer have a higher PSA density (when the prostate is controlled for volume) than Caucasians [8, 13]. However, the differences between African Americans and Caucasians in PSA velocity is controversial (assuming there is any difference at all). In one study, Caucasians had higher PSA velocities than African American men of a similar age, despite the fact that African Americans had a higher baseline PSA value, age-for-age [15]. This finding could be attributed to laboratory test access as perhaps African American men may have had fewer PSA tests over time, thus impacting their velocity score despite higher individual PSA values. Another study, reported that PSA velocity is higher in Caucasians only in the 60–69 age bracket, whereas in the 40–49 age group, PSA velocity is higher in African Americans. Nonetheless, despite the fact that PSA levels, density, and velocity appear different in African-American men compared to Caucasians, the USPSTF has issued guidelines that are not race specific.

### Mortality rate

An undeniable certainty is that regardless of previous screening recommendations, African American men have had a higher mortality rate from prostate cancer (two- to three-fold higher) and are carrying a larger prostate cancer burden than their Caucasian contemporaries [7]. African American men also are less likely to have health insurance and a higher proportion of them are of lower SES. In regards to SES, however, its impact on prostate cancer outcomes is controversial. For instance, Xu et al*.* reported that after adjusting for SES, the differences in prostate cancer mortality were eliminated. This is particularly relevant to screening because even among African Americans, screening guidelines may differ based on SES [4, 10, 11].

### USPSTF recommendation

In the past (2008), the USPSTF had stated there was insufficient evidence on the pros and cons of prostate cancer screening for men below age 75 (Grade I —insufficient knowledge of risks of the service), but that at and above age 75, screening should not be performed (Grade D — moderate or high risk of certainty that the service has no net benefit or the risks outweigh the benefits). The purpose of PSA screening is to reduce death rates directly linked to prostate cancer by intervening with treatment; however, screening and early treatment only benefited anywhere from 0 to 1 man per 1,000 men screened [16]. Currently, however, the USPSTF is against PSA based screening for male U.S. residents, regardless of ethnicity. It considers PSA screening to be “Grade D.” Prostate cancer has a lifetime risk of diagnosis estimated to be 15.9 %, while the lifetime risk of dying from prostate cancer is 2.8 % [2]. Thus, the argument is that many cases of prostate cancer are likely to have a favorable outcome and unlikely to impact survival, even without treatment or a delayed diagnosis.

Another USPSTF argument against screening is that even asymptomatic cancer is detected by PSA screening; often, these tumors will not progress or will progress so slowly that men are unlikely to experience deleterious symptoms in their lifetime. This type of overdiagnosis or pseudo-diagnosis is estimated to occur about 17 % to 50 % of the time in prostate cancer screening [16].

The USPSTF guidelines were based on two key studies: The Prostate, Lung, Colorectal, and Ovarian Cancer Screening Trial (PLCO) and the European Randomized Study of Screening for Prostate Cancer (ERSPC). According to the USPSTF, the PLCO Trial reported that only 4 % of enrolled men were non-Hispanic black while the ERSPC study, based on populations in seven countries with low populations of men of African descent, did not report demographic statistics. From that one can surmise that the majority of the subjects were likely Caucasian [16].

Several studies examining the utility of screening have generated differing findings. For example, the ERSPC tried to answer the question of whether screening using PSA leads to an improvement in cancer specific survival. Remarkably, it noted that at 11 years of follow-up, 1,055 additional men would need to be screened and 37 extra cases of prostate cancer would need to be detected to prevent just one death from prostate cancer [17].

In the PLCO Trial, the men who underwent annual prostate cancer screening with PSA and digital rectal examination had a 12 % higher incidence of prostate cancer than men in the control group [18]. The rate of death due to prostate cancer, however, was equal amongst both groups of men [18]. This study also found negative consequences such as pain and bleeding being associated with follow-up and treatment of abnormal screening tests [18].

The Prostate Cancer Intervention Versus Observational Trial (PIVOT) study compared expectant management to radical prostatectomy [19]. Its findings indicated that there were no differences in outcomes of prostate cancer in black men compared to white men [19]. Despite this finding, however, black men have higher rates of mortality due to prostate cancer. Moreover, only 30 % of its participants were identified as African American; hence, the trial was likely under-powered due to selection bias and is not generalizable to the African American community [19]. Although the general prostate cancer population as a whole may have a strong proportion that is being overtreated, African American men generally appear to be facing undertreatment and thus, the higher mortality rates.

### Molecular Markers

Evidence for differences between the course and outcome of prostate cancer in African American men also has been found in molecular markers. Several studies have been conducted regarding the location of SNPs and the differences in their rates of occurrence by race. Approximately 100 genes with SNPs are implicated in increasing men’s susceptibility to prostate cancer [20, 21]. These are especially accurate because SNPs are stable throughout one’s lifetime, so they cannot be affected by extraneous factors such as lifestyle [21]. Furthermore, several biomarkers appear to distinguish between aggressive and nonaggressive forms of prostate cancer; however, this information is not incorporated into conventional forms of prostate cancer screening [22]. The addition of genetic markers to current clinical parameters likely will detect the same number of prostate cancers without the need for a needle biopsy, making personalized medicine more effective and less invasive [23]. Unfortunately, African American men appear to be at an increased risk for developing prostate cancer in part because they possess several of these genetic variations. For instance, Xu et al*.* have reported that 17 of the 20 SNPs they have examined are more common in African American men and two of those 17 SNPs are associated with a higher risk of developing prostate cancer [24]. Other similar studies also have shown an association between African American men and specific genetic markers that appear to increase one’s susceptibility for developing prostate cancer. For example, Freedman et al*.*, noted there is a 3.8 MB interval on chromosome 8q24 that is associated with an increased risk of prostate cancer [25]. This chromosome interval is mainly found in African American men [25]. Individuals with a family history of allelic variants in this region are 9.46 times more likely to develop prostate cancer than someone without allelic variants [25].

Thus, despite the controversy associated with current prostate cancer screening, African American men need an updated set of screening guidelines that incorporate what is currently being used for screening with cutting edge molecular analyses to more successfully identify the patients who are at an increased risk of prostate cancer. Addressing the medical needs of such patients earlier will contribute significantly to reducing the burden of advanced disease, improve the chances of successful treatment, and lower the associated treatment costs.

### Other considerations

In 2013, the American Urological Association mentioned that African American race is considered to be a risk factor for prostate cancer, and that screening with PSA should be individualized based on personal preference and an informed discussion with health care providers after weighing the costs versus the benefits associated with screening [26]. We believe that this statement is not robust enough for African Americans. The American Urological Association’s stance has alluded to the fact that due to the difference in disease course, there ought to be some differences in the approach to screening for prostate cancer; however, we propose that this statement understates the necessity and importance of separate screening guidelines by failing to address the differences in disease course between African Americans and Caucasians.

The National Comprehensive Cancer Network and the American Cancer Society both also report that African American race is considered to be a risk factor for prostate cancer but they do not provide personalized screening guidelines [27, 28]. The American Cancer society goes one step further stating that African American men should begin discussions with their physician at age 45 (5 years earlier than men at average risk) [28]. However, neither the National Comprehensive Cancer Society nor the American Cancer Society have provided guidelines that meet the needs of African American men using an individualized approach.

It is also important to note that the lifetime risk of dying due to prostate cancer is approximately 3 % and men diagnosed with low to intermediate risk prostate cancer are unlikely to have a compromised lifespan due to said diagnosis [29]. Men with high risk prostate cancer do benefit from treatment if their projected life expectancy is greater than 10 years, but the majority of men who are diagnosed with high risk prostate cancer also have life expectancy of less than 10 years due to causes unrelated to their prostate cancer [27]. Hence, it is understandable that many individuals believe that prostate cancer is generally indolent and men who are diagnosed with prostate cancer often die of other causes unrelated to the cancer. However, it is our contention that this view is not generalizable to all prostate cancer patients as this data is derived from the entire male US population as opposed to considering just men of African American descent. Studies show that African American men with lower educational attainment have lower rates of prostate cancer screening; this discrepancy can in part be explained by a fatalistic attitude [30]. These fatalistic notions further highlight the importance of race specific guidelines for the African American community. By having separate screening guidelines, we can potentially overcome fatalism in African Americans regardless of their educational attainment and improve their survival.

It has been shown that African Americans have higher PSA levels in cancerous tumors [14, 31–35]. Even in non-cancerous prostates, African Americans have higher PSA levels [13, 36]. Furthermore, a large national cohort study found that African American veterans aged 40–54 were more likely to have 3 or more positive core biopsies yet they were less likely to be actively surveilled when compared to their white counterparts [36]. Indeed, African American veterans, aged 55–70, were more likely to have a higher grade and stage prostate cancer than African American veterans aged 40–54 [36]. There is new evidence, however, that highly undifferentiated tumors in African Americans may secrete less PSA [37]. This finding needs to be further explored, and the diversity of data available on this topic highlights the need for personalized guidelines.

Finally, it is crucial to consider that the current ad hoc approach to treatment of prostate cancer in African Americans is unreliable and needs to be better tailored. For example, Pietzak et al*.* found that the current criteria to evaluate which patients would benefit from active surveillance are not reliable in appropriately selecting African Americans when compared to Caucasians [38]. Kryvenko et al*.* found that over half of diagnosed prostate cancers considered to be insignificant (based on the Epstein criteria) were actually misclassified, in part due to a greater frequency of anterior tumors in African Americans compared to the tumors of white men [39]. Lastly, Sundi et al*.* have reported that African American men with very low risk prostate cancer at diagnosis still have a significantly higher prevalence of cancer at the anterior focus along with a higher grade and larger volume when compared to Caucasians [40].

## Conclusion

We have shown that the general prostate cancer screening guidelines developed by the USPSTF may be inappropriate for African American men because the course of the disease is different for them due to social and genetic characteristics. Furthermore, African American men appear to intrinsically have a higher incidence of prostate cancer and higher rates of mortality. It is important to note that currently there is no cure for metastatic prostate cancer and unfortunately, African American men are the most likely individuals to present with aggressive prostate cancer at initial diagnosis that advances to metastatic prostate cancer [7]. At the same time, there are subsets of African American patients who may not need aggressive treatment at all, similar to the fair portion of the general population diagnosed with prostate cancer. Both these groups of patients are not readily identified and thus, the screening recommendations have to be examined separately from treatment recommendations [41]. Given the above, we conclude that separate prostate cancer screening guidelines are important if we are to improve outcomes and save the lives of of African American men. At this time, we are working towards developing such guidelines by conducting a survey among national prostate cancer specialists. These guidelines will be the subject of future correspondence.

## Ethics approval and consent to participate

N/A.

## Consent for publication

N/A.

## Abbreviations

ERSPC: European Randomized Study of Screening for Prostate Cancer
PIVOT: Prostate Cancer Intervention versus Observational Trial
PLCO: Prostate, Lung, Colorectal, and Ovarian cancer screening trial
PSA: Prostate-Specific Antigen
SNP: Single Nucleotide Polymorphism
USPSTF: United States Preventive Services Task Force

## References

[1] Siegel RL, Miller KD, Jemal A (2015). Cancer Statistics 2015. CA Cancer J Clin.

[2] SEER Cancer Statistics Review, 1975–2006. National Cancer Institute Web site. http://seer.cancer.gov/archive/csr/1975_2006/ Published 2009. Accessed 01 June 2015.

[3] Odedina FT, Akinremi TO, Chinewundoh F, Roberts R, Yu D, Reams RR (2009). Prostate cancer disparities in Black men of African descent: a comparative literature review of prostate cancer burden among Black men in the United States, Caribbean, United Kingdom, and West Africa. Infect Cancer Agent.

[4] Behavioral Risk Factor Surveillance System Survey Data. Center for Disease Control and Prevention. http://www.cdc.gov/brfss/ Published 2009. Accessed 03 June 2015.

[5] Powell IJ, Heilbrun L, Littrup PL, Franklin A, Parzuchowski J, Gelfand D (1997). Outcome of African American men screened for prostate cancer: the Detroit Education and Early Detection Study. J Urol.

[6] Underwood W, De Monner S, Ubel P, Fagerlin A, Sanda MG, Wei JT (2004). Racial/ethnic disparities in in the treatment of localized/regional prostate cancer. J Urol.

[7] Powell IJ, Bock CH, Ruterbush JJ, Sakr W (2010). Evidence supports a faster growth rate and/or earlier transformation to clinically signifiant prostate cancer in black than in white American men, and influences racial progression and mortality disparity. J Urol.

[8] Henderson RJ, Eastham JA, Culkin DJ, Whatley T, Mata J, Venable D (1997). Prostate Specific Antigen (PSA) and PSA Density: Racial Differences in Men Without Prostate Cancer. J Natl Cancer Inst.

[9] McIntosh H (1997). Why Do African-American Men Suffer More Prostate Cancer?. J Natl Cancer Inst.

[10] Du XL, Fang S, Coker AL, Sanderson M, Aragaki C, Cormier JN (2006). Racial disparity and socioeconomic status in association with survival in older men with local/regional stage prostate carcinoma: findings from a large community-based cohort. Cancer.

[11] Ward E, Jemal A, Cokkinides V, Singh GK, Carinez C, Ghafoor A (2004). Cancer disparities by race/ethnic and socioeconomic status. CA Cancer J Clin.

[12] Whittemore AS, Kolonel LN, Wu AH, John EM, Gallagher RP, Howe GR (1995). Prostate cancer in relation to diet, physical activity, and body size in blacks, whites, and Asians in the United States and Canada. J Natl Cancer Inst.

[13] Abdalla I, Ray P, Ray V, Vaida F, Vijaykumar S (1998). Comparison of serum prostate-specific antigen levels and PSA density in African-American, white, and Hispanic men without prostate cancer. Urology.

[14] Vijayakumar S, Winter K, Sause W, Gallagher MJ, Michalski J, Roach M (1998). Prostatespecific antigen levels are higher in African-American than in white patients in a multicenter registration study: results of a RTOG 94–12. Int J Radiat Oncol Biol Phys.

[15] Preston DM, Levin LI, Jacobson DJ, Jacobson SJ, Rubertone M, Holmes E (2000). Prostate specific antigen levels in young white and black men 20–45 years old. Urology.

[16] Final Recommendation Statement: Prostate Cancer: Screening. U.S. Preventive Services Task Forcewebsite. http://www.uspreventiveservicestaskforce.org RecommendationStatementFinal/prostate-cancer-screening Published 2014. Accessed 04 June 2015.

[17] Schröder FH, Hugosson J, Roobol MJ, Tammela TL, Ciatto S, Nelen V (2012). Prostate-cancer mortality at 11 years of follow-up. N Engl J Med.

[18] Andriole GL, Crawford ED, Grubb RL, Buys S, Chia D, Church TR (2012). Prostate cancer screening in the randomized Prostate, Lung, Colorectal, and Ovarian Cancer Screening Trial: mortality results after 13 years of follow-up. J Natl Cancer Inst.

[19] Wilt TJ, Brawer MK, Barry MJ, Jones KM, Kwon Y, Gingrich JR (2009). The Prostate cancer Intervention Versus Observation Trial:VA/NCI/AHRQ Cooperative Studies Program #407 (PIVOT): design and baseline results of a randomized controlled trial comparing radical prostatectomy to watchful waiting for men with clinically localized prostate cancer. Contemp Clin Trials.

[20] Hicks C, Koganti T, Giri S, Tekere M, Ramani R, Sitthi-Amom J (2014). Integrative genomic analysis for the discovery of biomarkers in prostate cancer. Biomark Insights.

[21] Helfand BT, Catalona WJ, Xu J (2015). A genetic-based approach to personalized prostate cancer screening and treatment. Curr Opin Urol.

[22] David S, Chan DW (2014). Biomarkers in prostate cancer: what's new?. Curr Opin Oncol.

[23] Kader K, Sun J, Reck B, Newcombe PJ, Kim ST, Hsu FC (2012). Potential Impact of Adding Genetic Markers to Clinical Parameters in Predicting Prostate Biopsy Outcomes in Men Following an Initial Negative Biopsy: Findings from the REDUCE Trial. Eur Urol.

[24] Xu J, Kibel AS, Hu JJ, Turner AR, Pruett K, Zheng SL (2009). Prostate cancer risk associated loci in African Americans. Cancer Epidemiol Biomarkers Prev.

[25] Freedman ML, Haiman CA, Patterson N, McDonald GJ, Tadon A, Waliszewsk A (2006). Admixture mapping identified 8q24 as a prostate cancer risk locus in African-American men. Proc Natl Acad Sci U S A.

[26] Carter HB, Albertson PC, Barry MJ, Etzioni R, Freedland SJ, Greene KL et al. Early Detection of Prostate Cancer: AUA Guidelines. https://www.auanet.org/ Published 2013. Accessed 04 Mar 2016. 10.1016/j.juro.2013.04.119 PMC4020420 23659877

[27] National Comprehensive Cancer Network, Prostate Cancer. National Comprehensive Cancer Network website. http://www.nccn.org/patients/guidelines/prostate/ Published 2015. Accessed 03 Mar 2016.

[28] Prostate Cancer Prevention and Early Detection. American Cancer society website. http://www.cancer.org/cancer/prostatecancer/moreinformation/prostatecancerearlydetection/prostate-cancer-early-detection-acs-recommendations. Accessed 03 Mar 2016.

[29] Daskivich TJ (2015). Life Expectancy and Treatment Choice for Men with High-risk Prostate Cancer. Eur Urol.

[30] Hararah MK, Pollack CE, Garza MA, Yeh HC, Markakis D, Phelan-Emrick DF (2015). The Relationship Between Educational and Prostate-Specific Antigen Testing Among Urban African American Medicare Beneficiaries. J Racial Ethn Health Disparities.

[31] Vijayakumar S, Karrison T, Weichselbaum RR, Quadri SF, Awan AM (1992). Racial Differences in Prostate-specific Antigen Levels in Patients with Local-Regional Prostate Cancer. Cancer Epidemiol Biomarkers Prev.

[32] Vijayakumar S, Weichselbaum RR, Vaida F, Dale W, Hellman S (1996). Prostate-Specific Antigen Levels in African Americans Correlate with Insurance Status as an Indicator of Socioeconomic status. Cancer J Sci Am.

[33] Asbell S, Vijayakumar S (1997). Racial Differences in Prostate Specific Antigen Levels in Patients with Local-Regional Prostate Cancer. Prostate.

[34] Vijayakumar S, Vaida F, Weichselbaum R, Hellman S (1998). Race and the Will Rogers Phenomenon in Prostate Cancer. Cancer J Sci Am.

[35] Abdalla I, Ray P, Vijayakumar S (1998). Race and Serum Prostate-Specific Antigen Levels: Current Status and Future Directions. Semin Urol Oncol.

[36] Saltzman AF, Luo S, Scherrer JF, Carson KD, Grubb RL, Hudson MA (2015). Earlier Prostate-specific antigen testing in African American men—Clinical support for the recommendation. Urol Oncol.

[37] Kryvenko ON, Balise R, Soodana Prakash N, Epstein JI (2016). African American Men with Gleason Score 3 + 3 = 6 Prostate Cancer Produces Less Prostate Specific Antigen than Caucasian Men: A Potential Impact on Active Surveillance. J Urol.

[38] Pietzak EJ, Van Arsdalen K, Patel K, Malkowica SB, Wein AJ, Guzzo TJ (2015). Impact of Race on Selecting Appropriate Patients for Active Surveillance with Seemingly Low-risk Prostate Cancer. Urology.

[39] Kryvenko ON, Carter HB, Trock BJ, Epstein JI (2014). Biopsy Criteria for Determining Appropriateness for Active Surveillance in the Modern Era. Urology.

[40] Sundi D, Kryvenko ON, Carter HB, Ross AE, Epstein JI, Schaeffer EM (2014). Pathological Examination of Radical Prostatectomy Specimens in Men with Very Low Risk Disease at Biopsy Reveals Distinct Zonal Distribution of Cancer in Black American Men. J Urol.

[41] Adams LK, Ferrington LS (2014). New recommendations in prostate cancer screening and treatment. JAAPA.

